# Recognition of a Virtual Scene *via* Simulated Prosthetic Vision

**DOI:** 10.3389/fbioe.2017.00058

**Published:** 2017-10-10

**Authors:** Ying Zhao, Xiulin Geng, Qi Li, Guangqi Jiang, Yu Gu, Xiaoqi Lv

**Affiliations:** ^1^School of Information Engineering, University of Science and Technology, Baotou, China; ^2^School of Computer Engineering and Science, Shanghai University, Shanghai, China

**Keywords:** prosthetic vision, virtual scene, pathfinding, object identification, collision detection

## Abstract

In order to effectively aid the blind with optimal low-resolution vision and visual recovery training, pathfinding and recognition tests were performed using a simulated visual prosthetic scene. Simple and complex virtual scenes were built using 3DMAX and Unity, and pixelated to three different resolutions (32 × 32, 64 × 64, and 128 × 128) for real-time pixel processing. Twenty subjects were recruited to complete the pathfinding and object recognition tasks within the scene. The recognition accuracy and time required were recorded and analyzed after the trials. In the simple simulated prosthetic vision (SPV) scene, when the resolution was increased from 32 × 32 to 48 × 48, the object recognition time decreased from 92.19 ± 6.97 to 43.05 ± 6.08 s, and the recognition accuracy increased from 51.22 ± 8.53 to 85.52 ± 4.93%. Furthermore, the number of collisions decreased from 10.00 ± 2.31 to 3.00 ± 0.68. When the resolution was increased from 48 × 48 to 64 × 64, the object recognition time further decreased from 43.05 ± 6.08 to 19.46 ± 3.71 s, the recognition accuracy increased from 85.52 ± 4.93 to 96.89 ± 2.06%, and the number of collisions decreased from 3.00 ± 0.68 to 1.00 ± 0.29. In complex scenes, the time required to recognize the room type decreased from 115.00 ± 23.02 to 68.25 ± 17.23 s, and object recognition accuracy increased from 65.69 ± 9.61 to 80.42 ± 7.70% when the resolution increased from 48 × 48 to 64 × 64. When the resolution increased from 64 × 64 to 128 × 128, the time required to recognize the room type decreased from 68.25 ± 17.23 to 44.88 ± 9.94 s, and object recognition accuracy increased from 80.42 ± 7.71 to 85.69 ± 7.39%. Therefore, one can conclude that there are correlations between pathfinding and recognition. When the resolution increased, the time required for recognition decreased, the recognition accuracy increased, and the number of collisions decreased. Although the subjects could partially complete the recognition task at a resolution of 32 × 32, the recognition time was too long and recognition accuracy was not good enough to identify simple scenes. Complex scenes required a resolution of at least 48 × 48 for complete recognition. In addition, increasing the resolution shortened the time required to identify the type of room, and improved the recognition accuracy.

## Introduction

When people see, light in the visible spectrum is reflected by the objects in the environment, passes through the corona, is focused by the lens, and forms images on the retina. Then, the visual nerve system delivers the information to the brain, where it is perceived as vision. Through the visual system, humans and animals perceive object sizes, brightnesses, colors, and movement and obtain various types of information. At least 80% of external information is obtained *via* vision; it is the most important human and animal sense (Haibin, 2011; Weizhen, 2013).

According to statistical reports on visual impairment and blindness by the World Health Organization, 285 million people are vision impaired worldwide. Of these, 39 million are blind and 246 million suffer from amblyopia. About 90% of the world’s vision impaired people live in low income countries, and 82% of blind patients are at least 50 years old. Since the world’s population is aging, this number is expected to rise (World Health Organization, 2009). The main causes of blindness are cataracts (Javitt et al., 1991) and age-related macular degeneration (AMD) (Jager et al., 2008). Although treatment for cataracts is available, AMD has no effective treatment and is one of the main causes of blindness (Chunyong, 2010). Macular degeneration is a chronic, incurable disease that can cause a sharp decline in central visual acuity and led to irreversible decreases in, or loss of central vision. Central vision is necessary for reading, determining the time, recognition of facial features, driving, and other daily activities (Shaohua and Yu, 2015). Macular degeneration usually occurs among those over 45 years old, and its incidence increases with age. The incidence of macular degeneration depends on race, with the disease being more common among whites than among colored people (Elfervig, 1998).

Other causes of blindness include primary retinitis pigmentosa (RP) (Senthil et al., 2017), congenital blindness (Greenaway and Dale, 2017), corneal opacity (Durkin et al., 2007), uncorrected refractive errors (Naidoo and Jaggernath, 2012), and diabetic retinopathy (Busik et al., 2017). The historically common retinal pigment degeneration called RP is frequently observed in people with underlying retinal degeneration (Senthil et al., 2017). According to survey data, its prevalence in some parts of China is about 1 in 3,500 (Shintani et al., 2009). The disease manifests as chronic, progressive retinal degeneration, eventually leading to blindness. The disease in some RP patients are autosomal dominant, which means that if one parent has the pathogenic gene, then the children will have the disease. However, some of them is linkage inheritance, which means if the mothers carry the disease genes, their children will be sick. Other cases may be accompanied by hearing loss and more common among males (Hongmin, 2004).

The blind person who lives in the darkness bears pain that sighted people cannot understand (Menghui and Qiushi, 2015). The birth of visual prostheses has given blind people the hope of recovering eyesight but is not applicable to all types of blindness. Currently, visual prostheses are designed primarily for RP and AMD patients (Kawashima et al., 2015). In these cases, the cause of blindness is apoptosis of retinal lateral photoreceptor cells and loss of the ability photosensitive. The ganglion and bipolar cells in the inner retina are still alive. Most of these surviving neurons can be stimulated by visual prostheses, resulting in artificial vision. Although the resulting visual acuity is far lower than normal, even such low-resolution vision serves as a “light” for the blind to save them from a dark world (Srivastava et al., 2009; Menghui and Qiushi, 2015).

In principle, visual prostheses use micro cameras to obtain images, process and encode them using an image processor, and transmit the information through wireless coils to a micro stimulator implanted in the body. This device receives the information, decodes it and sends signals to a microelectrode array that stimulates the visual nervous system to induce luminescence photoism, resulting in artificial vision (Duret et al., 2006; Chader et al., 2009; Rizzo, 2011; Zapf et al., 2015).

Although the principle is simple, it is very difficult to accomplish artificial vision. First, the structure of the eye and the construction of the visual cortex are very fine and provide a limited operating area. The prosthesis requires implanting dozens or even hundreds of electrodes within the macular area of 5 mm in diameter (Jinhai et al., 2007; Seungwoo et al., 2009). Furthermore, compatibility requirements for biological materials are high. In addition, the mechanisms of the human brain and visual neural system are not yet fully understood. Since it is still unclear how the brain perceives and processes color through vision, current visual prostheses only present “black and white” vision formed by phosphenes.

The United States, Germany, Australia, Belgium, Japan, etc. have carried out research on and designed visual prostheses. Some researchers have reported clinical and commercial applications. In the United States, Second Sight produces an epiretinal prosthesis called Argus II (Barry et al., 2012; Cruz et al., 2013), which was authorized by the European EMA in 2011 and the United States FDA in 2013 (Stronks and Dagnelie, 2014). Alpha-IMS, a subretinal prosthesis by Retina Implant AG in Germany won European EMA authorization allowing it to begin sales in 2013 (Stingl et al., 2013). In addition, Australia’s Vitoria Monash University Clayton campus researchers have developed a bionic vision system called “bionic eye” that bypasses the visual pathway and directly stimulates the primary visual cortex *via* a series of 9 mm × 9 mm tiles implanted into the brain to let patients perceive visual patterns from combinations of up to 473 spots of light (phosphenes). Although perception from the normal eye produces about 1,500,000 pixels, the rough, artificial image allows users to identify objects with simple shapes, the directions of moving objects, and perform other daily tasks (Kaufman, 2016).

To improve object identification and performance, a simulated prosthetic visual test was conducted with sighted people to evaluate the potential benefits of particular electrode arrays. The results can provide useful aid to clinical studies (Dagnelie et al., 2007; Chen et al., 2009; Zhao et al., 2010). In this article, a virtual scene was created using 3DMAX and Unity and presented to the subjects after binarization, color inversion, and simulated phosphenes template matching. The subjects used the first-person view and were asked to use the real-time pixelated scene to perform pathfinding and object recognition tasks by using the mouse and WASD (or arrow) keys on a keyboard to control their orientations and directions of movement. The object identification time, object recognition accuracy, and the types and occurrences of collisions were detected and analyzed at various resolutions. We hope the results of this work will provide effective guidance and help in the development of image processing strategies, visual prosthesis stimulation, and visual recovery training, as well as further guidance on prosthesis implantation in patients with non-terminal RP.

## Materials and Methods

### Selection of Subjects

Twenty volunteers were recruited from the Inner Mongolia University of Science and Technology graduate school. They had normal or corrected visual acuities of 20/20. The subjects were between 22 and 29 years old, with half being men and half women.

### Compliance Statement

All of the experimental process meet the requirements of the Helsinki declaration of the World Medical Association and comply with national medical device clinical trial requirements. All participating subjects were informed of the course and purpose of the experiment and signed informed consent documentation before participation. According to the local and national guidelines, this study was not required specifically reviewed and approved by an ethics committee.

### Equipment and Environment

The experimental platform included a camera (Logitech c920) and two personal computers. Software such as Audodesk 3ds Max 2012, Unity 4.3.4f1, Statistical Product and Service Solutions (SPSS), Java, C #, Visual Studio 2010, Matlab 2014b, screen video expert V2016, etc. were used to make models, as well as to perform image processing and statistical analysis.

The data points represent the mean value (seconds or accuracy ± SD) for combined data from all subjects. Data were analyzed using ANOVA and *t*-tests (two-tailed; a Bonferroni correction was applied to multiple comparisons) with SPSS 19.0 for Windows (SPSS Inc.), and *p* values ≤ 0.05 (after correction) were considered significant.

**Figure 1:**
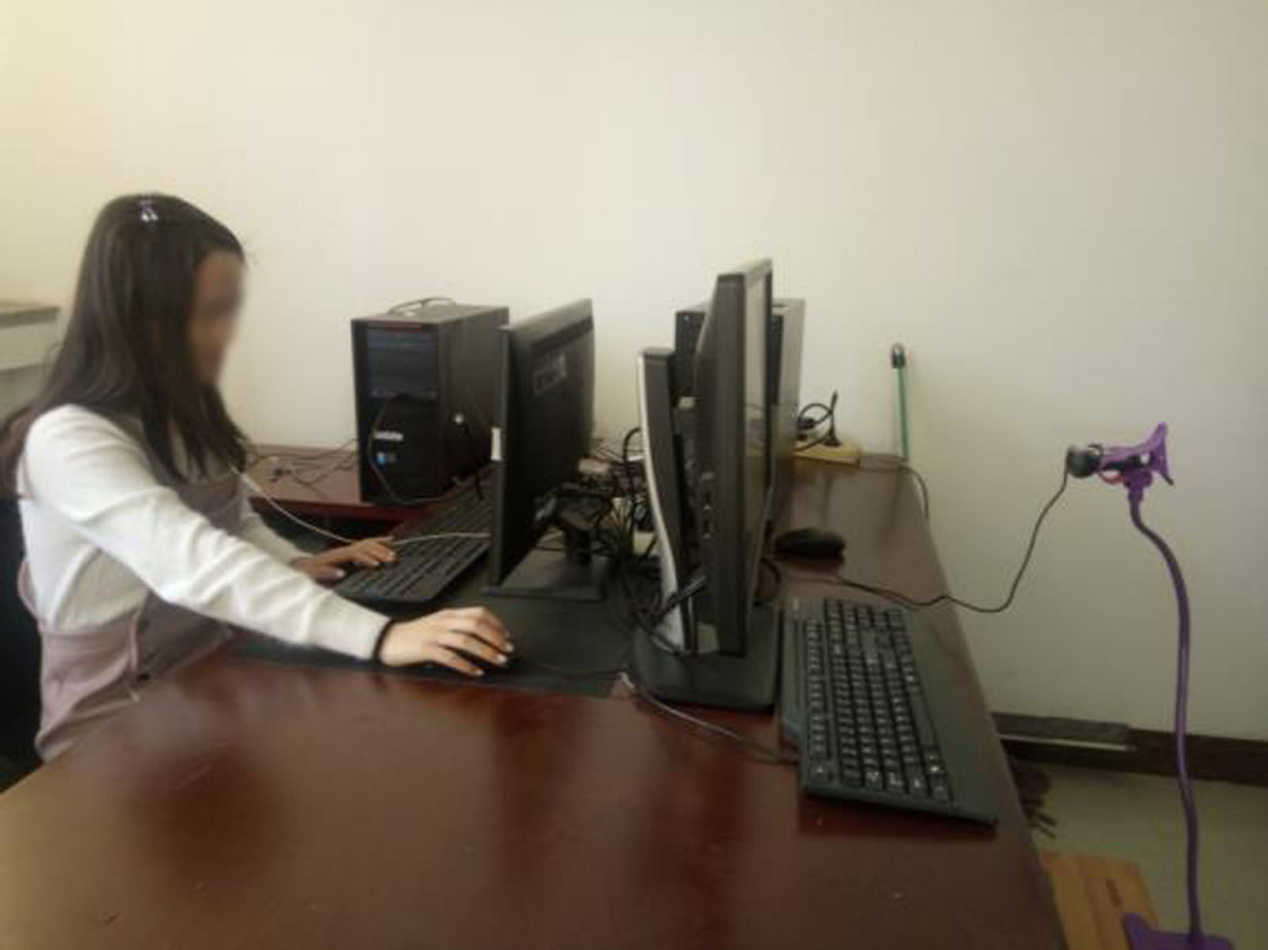
Photograph of a specific experimental scenario.

Before the experiments began, we ensured that the participants experienced no interruptions and remained relaxed. The subjects sat in front of the LCD monitor at a distance of 40–50 cm. One of the computers with a screen resolution of 1,920 × 1,080 ran a real-time pixelization program that captured the virtual scene information from the camera and processed it into pixelated images. The virtual scene was built in Unity 4.3.4f1, which was running on another desktop station. Subjects were asked to use the direction keys on the keyboard to control movement in the scene and walking speed. The mouse was used to control the angle of vision, and audio prompt information was provided through the headset. Figure 1 shows the experimental scenario.

### Material Library and Preexperiment Data

Because of individual differences in operation of the mouse and keyboard in a virtual environment with first-person perspective, the subjects with a good sense of direction, or who are familiar with first-person video games or the housing layout were filtered and recruited *via* a questionnaire. Then, the preexperimental training were performed.

The scenarios used in preexperimental training included a model that was created in 3DMAX and transferred into the unity virtual scene. Collision processing and first-person perspective were added, and the mouse and keyboard were configured to control viewing and ensure that the walking speed was in line with a normal pace. The default line of sight was horizontal and set to 0°. The vertical plane view was set to 50° above and 70° below this, and the horizontal field of view was about 50° from left to right. These settings placed the upper, lower, left, and right perspectives in line with those normally provided by the human eye (Shizhong, 1996).

Collision detection was used to cause doors to open automatically when users approached the door handles. Sound prompts were set to activate when people identified or collided with objects or the wall, or entered the door. The type of interaction, door, and time were recorded in the software. Next, the entire virtual scene was binarized and placed in four different pixelated templates with the following resolutions: f32 (32 × 32), f48 (48 × 48), f64 (64 × 64), and f128 (128 × 128), to produce real-time, pixelated virtual scenes.

**Figure 2:**
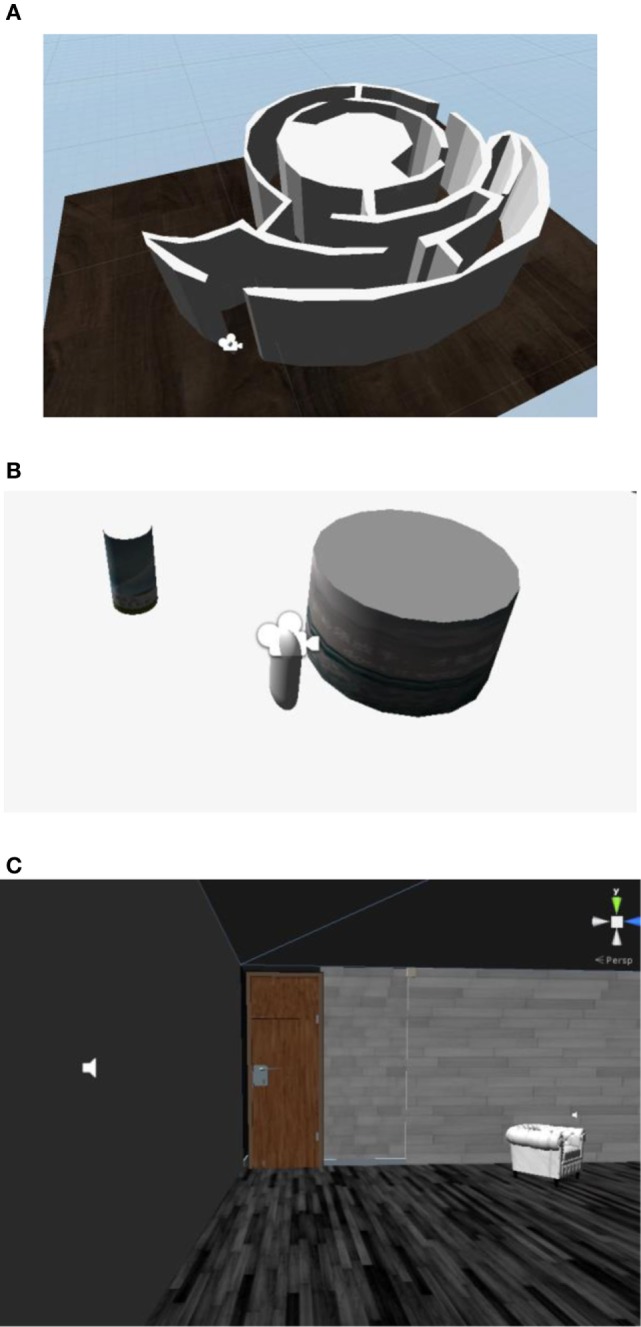
Preexperimental scenarios in the first-person perspective. **(A)** Preexperiment scene I: maze walking. **(B)** Preexperiment scene II: cylindrical literacy. **(A,B)** show scene layouts before operation, with the camera showing the user’s perspective; **(C)** shows a running scene in which the subjects used the first-person point of view to see specific information in the scene. The speaker provides audible feedback after collisions.

The preexperimental scene required three tasks:

1. A pathfinding task called maze walking. Subjects controlled the perspective and the direction of movement, and used the mouse and direction keys to complete the maze.
2. An identification task called cylindrical literacy. Subjects identified the text on nearby large and small cylinders.
3. Way-finding and identification tasks. In simple indoor scenes, subjects were required to find and identify the sofa and the door. They needed to open the door and complete pathfinding tasks at the same time. It was necessary for them to become familiar with the collision alarm and the sound of the door switch. The specific preexperiment scenario is shown in Figure 2. After the preexperiment, the subjects could use the equipment independently, respond to the alarm and the sound of the door, and conduct normal activities in the virtual environment.

### Formal Experiments

**Figure 3:**
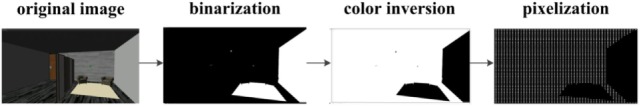
The scene pixelization process.

Formal experiments were carried out using real-time scene processing. The first computer used Unity to present a virtual scene. The real-time video image was captured by a camera connected to the second computer, binarized, inverted, pixelated, and presented to the subjects. The subjects controlled the mouse and keyboard to perform specified tasks. Figure 3 shows the scene pixelization process.

There were two scenarios that had different tasks in the formal tests. In the pathfinding situation, the first scene (scene I) was composed by several rooms with a corridor. Along the corridor, two offices containing various items were located on one side. The doors were configured for collision detection and could open or shut automatically when the user approached or entered a room. Furthermore, there were audible responses when users collided with walls or objects.

The task was to perform pathfinding and identification with assistance from door opening/closing sounds. In addition, collisions were detected. The subjects were required to identify the door positions with the previously described audible assistance and perform object recognition in the hall and the room. They were informed in advance of the need to find the remaining two doors and encouraged to identify the object, the door, and the door handle position by walking and changing perspectives. The time allotted for object identification was limited to 2 min.

The time required to enter the door included the time required to identify the door, approach the door, identify the door handle, and complete the entry. The exit time included identifying the door, approaching the door, and actuating the door handle to exit.

**Figure 4:**
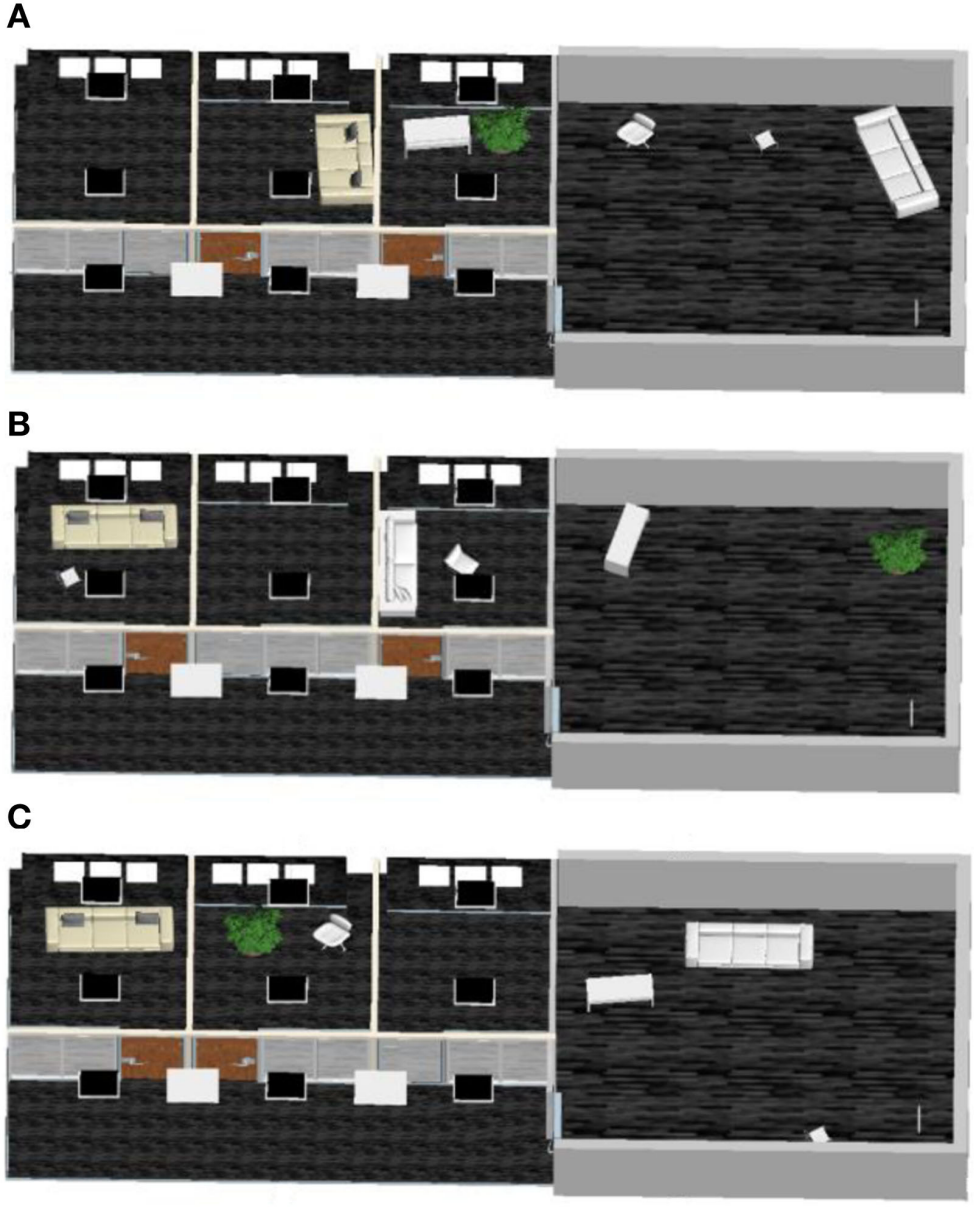
Schematic diagram of formal experiment scene I. **(A–C)**, respectively, denote the transformed scene at f32, f48, and f64 resolutions.

The recognition time, accuracy, and collision information (including type, time, and frequency) were recorded at each resolution by the experimenter. In order to prevent learning effects, the shapes, sizes, and locations of the objects in the hall and offices were different in each of the three resolutions, as shown in Figure 4.

The second scene (scene II) included a house with two bedrooms, two bathrooms, a study, a kitchen, a dining room, and a living room. In the second scene, the task was to complete pathfinding and identification. The dining room and living room were connected and all of the doors were open. In the pathfinding situation, subjects had to walk in a straight line and turn in the house. They had to determine room types based on their life experiences. The subjects who more effectively determined the room types would continue to complete the following four tasks:

1. Find the bedroom lamp.
2. Find the kitchen pot.
3. Find the bathroom toilet.
4. Find the toilet in another bathroom.

**Figure 5:**
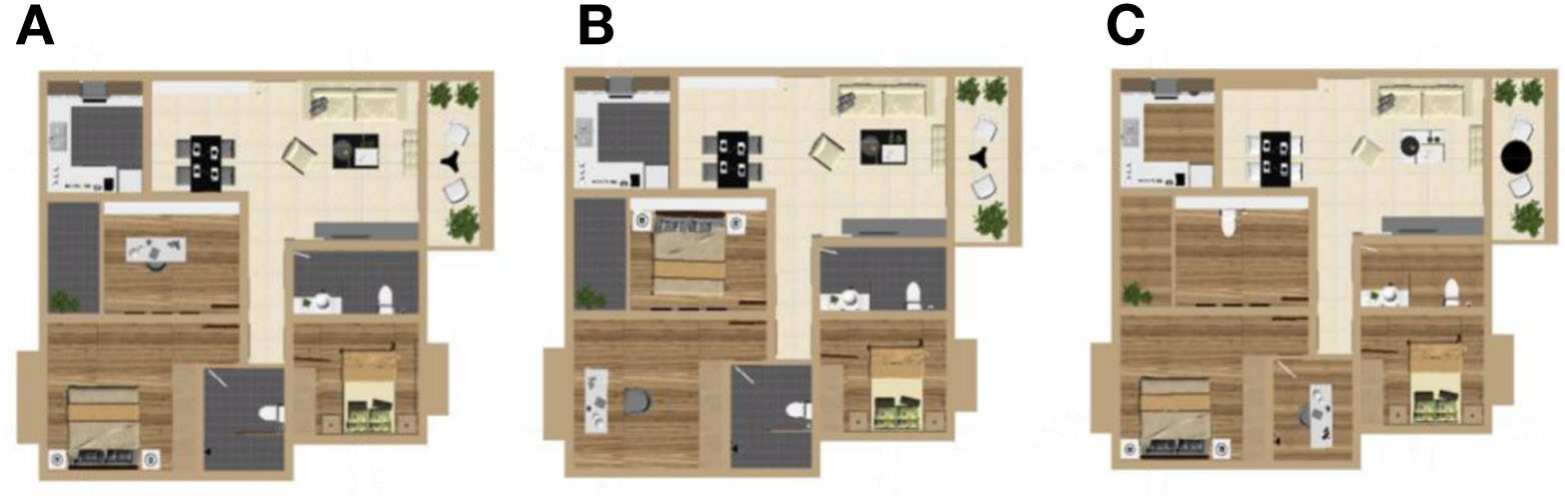
Schematic diagram of formal experiment scene II. **(A–C)**, respectively, expressed the transformed scene at f32, f48, and f64 resolutions.

In order to prevent learning effects, the locations of the rooms and the shapes, sizes, and locations of the objects were different in each of the three resolutions, as shown in Figure 5. There were no sound effects, the subjects were informed in advance not to move in the event of a collision. All of the doors were open and subjects were asked not to go to the rear door. The recognition time and accuracy under different resolutions were recorded by the experimenter.

Before the experiment, the subjects were encouraged not to look at the ground and try to face forward to keep horizontal eye level. They were informed that the door locations would be different in each of the three resolutions and that they should determine the specific type of object according to the object’s height, size, and shape.

## Results

### Analysis of the First Scene

**Figure 6:**
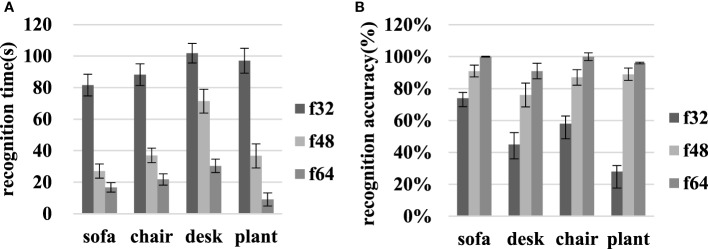
Object recognition time and identification accuracy for the sofa, chair, desk, and green plant in scene I at various resolutions. **(A)** Recognition time, and **(B)** accuracy.

As Figure 6A shows, the identification times for the sofa, chair, desk, and green plant decreased from 81.60 ± 6.82, 88.25 ± 6.94, 101.80 ± 6.24, and 97.10 ± 7.89 to 16.65 ± 2.96, 21.80 ± 3.57, 30.35 ± 4.21, and 9.05 ± 4.11 s, respectively, as the resolution increased from f32 to f64. Therefore, the average identification time decreased from 92.19 ± 6.97 to 43.05 ± 6.08 and 19.46 ± 3.71 s at f32, f48, and f64. Using the *t*-test, significant differences were found within f32–f48 and f48–f64 resolution comparisons (*p* < 0.05).

On transitioning from f32 to f64, the accuracy with which the sofa, chair, desk, and green plant were identified increased from 73.75 ± 5.38, 45.00 ± 9.02, 58.33 ± 9.39, and 27.78 ± 10.32 to 100.00 ± 0.30, 91.45 ± 4.93, 100.00 ± 2.50, and 96.11 ± 0.50%, respectively as Figure 6B shows. Then, the average accuracy increased from 51.22 ± 8.53 to 85.52 ± 4.93 and 96.89 ± 2.06% at f32, f48, and f64. The recognition accuracy improvements associated with the f32–f48 and f48–f64 comparisons were significant for each object (*p* < 0.05).

**Figure 7:**
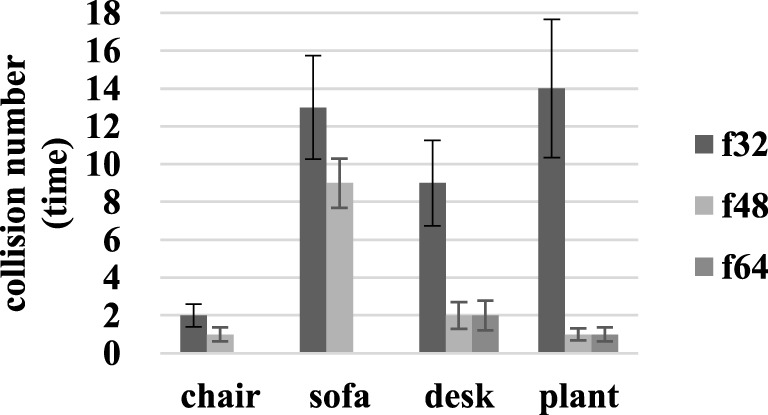
Collision detection results during identifying the chair, sofa, desk, and green plant.

In addition, collisions were detected and recorded. As shown in Figure 7, there were 2.00 ± 0.59, 1.00 ± 0.38, and 0 ± 0 collisions at f32, f48, and f64 resolutions during chair identification. The number of collisions associated with finding the sofa decreased from 13.00 ± 2.73 to 9.00 ± 1.31 and 0 ± 0 when the resolution increased from f32 to f48 and f64. During the process of identifying the desk, 9.00 ± 2.26 collisions occurred at f32, while 2.00 ± 0.71, 2.00 ± 0.78 collisions occurred at f48 and f64. Plant identification led to 14.00 ± 3.64 collisions at f32 and 1.00 ± 0.38, 1.00 ± 0.32 collision at f48 and f64. The average collisions decreased from 10.00 ± 2.31 to 3.00 ± 0.68 and 1.00 ± 0.29 at f32, f48, and f64.

**Figure 8:**
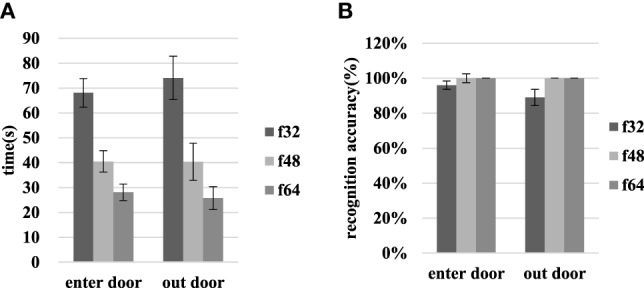
The time requirements and recognition accuracies for entry and exit through the door in scene I at various resolutions. **(A)** Shows the time required and **(B)** shows the recognition accuracy.

As shown in Figure 8, the time required to enter the door decreased from 68.05 ± 5.74 to 28.10 ± 3.29 s, and the time required to exit decreased from 74.05 ± 8.71 to 25.75 ± 4.64 s when the resolution increased from f32 to f64. The paired sample *t*-test based on entry and exit data for different resolutions indicates significant differences associated with the f48–f64 and f32–f48 resolution comparisons.

When the resolution increases from f32 to f48, the door entry recognition accuracy rate increases from 96.30 ± 2.34 to 100.00 ± 0%, and the door exit recognition accuracy rate increases from 88.89 ± 4.65 to 100.00 ± 0%. At f48 and f64, the recognition accuracy associated with entering or exiting through the door reaches 100%. The results of the *t*-test that compares door entry/exit at different resolutions are *p* > 0.05.

### Analysis of the Second Scene

**Figure 9:**
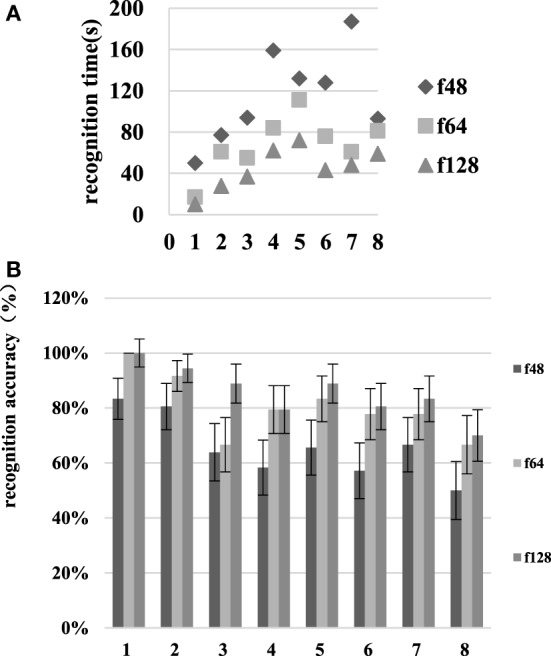
Room identification time and recognition accuracy at various resolutions in scene II. The numbers 1–8 on the abscissa represent the living room, dining room, kitchen, study, bedroom one, bedroom two, bathroom one, and bathroom two, respectively. **(A)** is the recognition time and **(B)** is the recognition accuracy.

As is seen in Figure 9, as the resolution increases from f48 to f64, the room type recognition time decreases from 115.00 ± 23.02 to 68.25 ± 17.23 s, and the recognition accuracy rate increases from 65.69 ± 9.61 to 80.42 ± 7.70%. When the resolution increases from f64 to f128, the room recognition time decreases from 68.25 ± 17.23 to 44.88 ± 9.94 s, and the recognition accuracy increases from 80.42 ± 7.70 to 85.69 ± 7.39%. The recognition time and accuracy for the three types of room were tested using the paired sample *t*-test. The identification times changed significantly (*p* < 0.05) in the f48–f64 and f64–f128 comparisons. The recognition accuracy was significantly different for the f48–f64 comparison, but there was no significant difference associated with the f64–f128 comparison.

**Figure 10:**
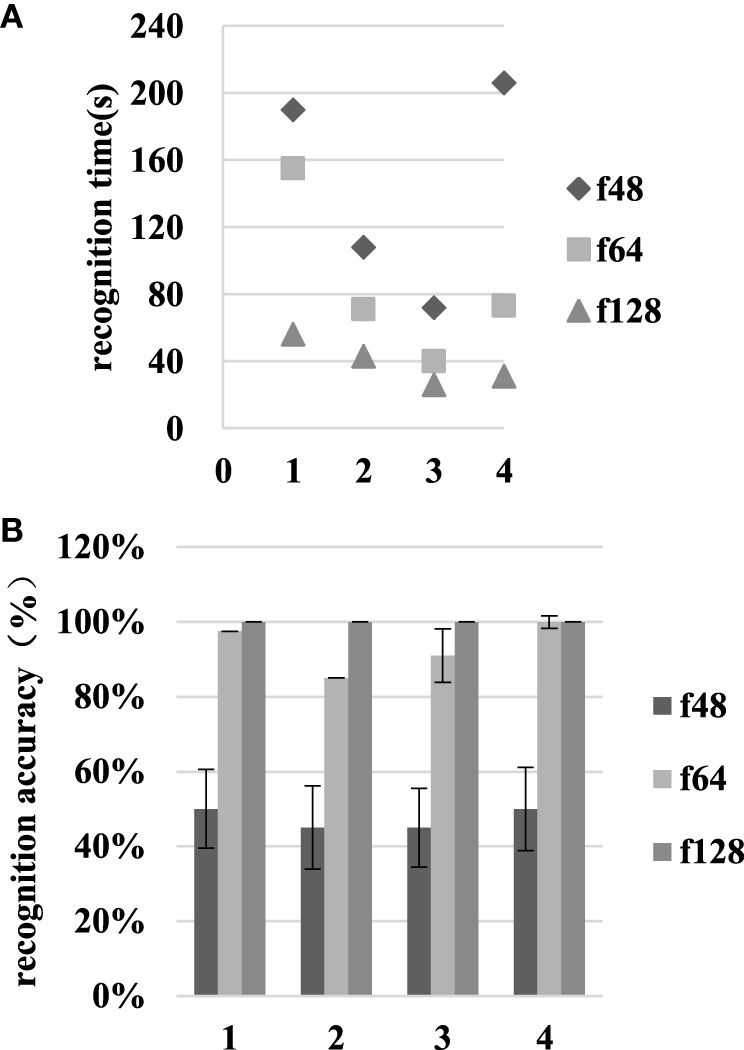
The recognition time and accuracy results at various resolutions in scene II. The numbers 1–4 on the abscissa represent tasks 1–4, respectively. **(A)** is the recognition time and **(B)** is the recognition accuracy.

As Figure 10 shows, as the resolution increases from f48 to f64, the recognition time for task completion decreases from 144.00 ± 22.23 to 84.75 ± 16.11 s, and the recognition accuracy increases from 47.50 ± 10.86 to 93.38 ± 2.21%. When the resolution increases from f64 to f128, the room type recognition time decreases from 84.75 ± 16.11 to 39.00 ± 6.13 s, and the recognition accuracy increases from 93.38 ± 2.21 to 100.00 ± 0%. The *t*-test shows significant recognition time differences in the f48–f64 and f64–f128 resolution comparisons. There are significant recognition accuracy differences in the f48–f64 comparison, but no significant differences in the f64–f128 comparison.

The subjects selected to participate in the scene II task performing experiment had better performance in scene I and scene II identifications. Familiarity with the test environment still caused a significant difference in the results, but the same trends emerged, with the f64 resolution effectively applicable to the default identification task.

## Discussion and Conclusion

### Advantages of Virtual Scenes

Virtual scenes can be built to implement the intended experimental platform without constraints associated with real scenes or other factors. Furthermore, use of virtual scenes can help to avoid the experimental error caused by collisions and the use of touch to identify objects indirectly. Because the virtual scene is artificial, we selected subjects who play virtual video games frequently and were familiar with scenes similar to ours *via* a questionnaire. Before the formal experiment, preexperimental training was carried out to familiarize subjects with the experiments and input device operation. The experimenter was able to improve the experimental design using feedback from the subjects.

### Resolution Selection

In experimental scene I, the virtual experimental scene was pixelated into three resolutions (f32, f48, and f64). Before the formal test, we tested an f16 (16 × 16) resolution scene. It was difficult to identify the locations and directions of the objects, as well as to control the mouse and keyboard for pathfinding and identification. Although the results were not as good as with the actual scene in f64 resolution, the subjects could complete pathfinding and identification. In the more complex scene II, the subjects were asked to identify the type of room by identifying the items in it. Although each room was distinctive, it was necessary to identify at least one or two items in the room and to control the approximate direction of travel. In the preexperiment, subjects were unable to perform pathfinding and room type identification at f32 resolution. Hence, f48, f64, and f128 were selected for comparison in the complex scenario of scene II.

### Individual Difference

In the identification task in scene I, 1 of the 20 subjects identified the sofa in 2.5 s at f48 resolution and in 15 s at f64. A similar situation occurred in green plant identification, where the identification time was 18 s at f32 and 22 s at f48. These events may have occurred because the subjects did not respond to changes in object locations in different scenes of various resolutions. With regard to the door entry process, the recognition times of the two subjects increased upon the transition from f48 to f64. This may be due to the change in door position between the different scenes. Although we hinted at the position changes before the formal test, some subjects failed to act carefully, resulting in experimental error. In the process of exiting through the door, one subject’s recognition time increased from f32 to f48 and the other subject’s recognition time increased from f48 to f64. The subject recognized the door hinge as the door handle while exiting, and was unable to exit from the door hinge position. This may have led to fluctuations in the results of the experiment.

### Conclusion

In the relatively simple simulated visual scene, only a subset of the targeted pathfinding and identification tasks could be completed at f32 resolution. As the resolution increased, the recognition time decreased and the recognition accuracy improved. For identification, increasing the resolution significantly increases the recognition accuracy. Particularly significant changes occurred between f32 and f48. Subjects could enter and exit through the door with recognition accuracy of 100% at f48. Changing the resolution from f48 to f64 had no significant effect on completion of the task. In addition, collision detection mechanisms were configured for the four objects. The collisions were counted at each resolution after completion of identification. As the resolution increased, the number of collisions gradually decreased. Subjects could identify the objects without collisions at f64.

In the more complex simulated visual scene, subjects could partially complete the same pathfinding and identification tasks at f48. As the resolution increased, the time required to identify the room type was shortened and the recognition accuracy increased. The recognition times associated with the f48–f64 and f64–f128 resolution pairs exhibited significant differences, and the recognition accuracy changed significantly between f48 and f64. No significant difference was noted between f64 and f128. Thus, a resolution of f64 is appropriate for simple pathfinding and identification tasks in the complex simulation scene.

We hope that these conclusions provide meaningful guidance that helps blind people to benefit from the best low-resolution visual technologies. We also hope that they can be used to aid in subsequent physiological information acquisition and analysis, and for further studies.

## Ethics Statement

All the experimental process meet Helsinki declaration of the World Medical Association, compliance with national medical device clinical trials. All participating subjects were informed of the course and purpose of the experiment and signed informed consent before participation.

## Author Contributions

ZY designed the experiments and wrote this article. GXL performed the experiments and analyzed the experimental results. LQ and JGQ performed the experiments. GY and LXQ designed the experiments and provided modification suggestion.

## Conflict of Interest Statement

The authors declare that the research was conducted in the absence of any commercial or financial relationships that could be construed as a potential conflict of interest.
